# The prognostic value of neutrophil-to-lymphocyte ratio in cholangiocarcinoma: a systematic review and meta-analysis

**DOI:** 10.1038/s41598-022-16727-w

**Published:** 2022-07-25

**Authors:** Dong Liu, Lara R. Heij, Zoltan Czigany, Edgar Dahl, Marcel den Dulk, Sven A. Lang, Tom F. Ulmer, Ulf P. Neumann, Jan Bednarsch

**Affiliations:** 1 Department of Surgery and Transplantation, University Hospital RWTH Aachen, Pauwelsstrasse 30, 52074 Aachen, Germany; 2 Institute of Pathology, University Hospital RWTH Aachen, Aachen, Germany; 3 Department of Surgery, Maastricht University Medical Center (MUMC), Maastricht, The Netherlands

**Keywords:** Prognostic markers, Surgical oncology

## Abstract

The neutrophil-to-lymphocyte ratio (NLR) is used as biomarker in malignant diseases showing significant association with poor oncological outcomes. The main research question of the present study was whether NLR has also prognostic value in cholangiocarcinoma patients (CCA). A systematic review was carried out to identify studies related to NLR and clinical outcomes in CCA evaluating the literature from 01/2000 to 09/2021. A random-effects model, pooled hazard ratios (HR) and 95% confidence interval (CI) were used to investigate the statistical association between NLR and overall survival (OS) as well as disease-free survival (DFS). Subgroup analyses, evaluation of sensitivity and risk of bias were further carried out. 32 studies comprising 8572 patients were eligible for this systematic review and meta-analysis. The pooled outcomes revealed that high NLR prior to treatment is prognostic for poor OS (HR 1.28, 95% CI 1.18–1.38, p < 0.01) and DFS (HR 1.39, 95% CI 1.17–1.66, p < 0.01) with meaningful HR values. Subgroup analysis revealed that this association is not significantly affected by the treatment modality (surgical vs. non-surgical), NLR cut-off values, age and sample size of the included studies. Given the likelihood of NLR to be prognostic for reduced OS and DFS, pre-treatment NLR might serve as a useful biomarker for poor prognosis in patients with CCA and therefore facilitate clinical management.

## Introduction

Cholangiocarcinoma (CCA) accounts for 15% of all primary malignant liver tumors and arises from intra- or extrahepatic bile ducts^1,2^. Due to the anatomical location of the tumor in the extrahepatic (extrahepatic CCA, ECCA) subtype and the usually higher tumor burden in the intrahepatic subtype (intrahepatic CCA, ICCA), clinical outcomes have been reported to be dismal even after radical surgery in comparison to other gastrointestinal tumors^3–5^. Therefore, the identification of reliable prognostic markers might facilitate patient selection as well as risk-stratification in CCA patients.

Inflammation in the tumor microenvironment plays a well-known and important role in tumor biology. Particularly, carcinogenesis and tumor progression are often linked to systemic inflammatory activation^6^. Over the past years, several prognostic scores on the basis of laboratory parameters, such as the counts of neutrophiles, lymphocytes as well as C-reactive protein (CRP) levels, have been developed. Based on this, calculated scores e.g. neutrophil-to-lymphocyte ratio (NLR), platelet-to-lymphocyte ratio (PLR), and Glasgow Prognostic Score (GPS), have been frequently associated with oncological outcomes in various solid tumors^7–10^. However, conflicting results have been reported regarding the prognostic value of these preoperative systemic inflammatory parameters in CCA^11–13^.

Given the prognostic value in other tumor entities, it is hypothesized that NLR has also prognostic value in CCA. Thus, a systematic review and meta-analysis is conducted to further assess the prognostic value of NLR for oncological outcome [overall survival (OS), disease-free survival (DFS)] in CCA patients based on the available evidence.

## Material and methods

### Literature search

The ex-ante protocol of this systematic review was registered open access in the International Prospective Register of Systematic Reviews (PROSPERO) under the ID: CRD42021271435 and was conducted in line with recommendations of the PRISMA (Preferred Reporting Items for Systematic Reviews and Meta-analyses) statement. PubMed and Google Scholar were systematically searched for articles published between January 2000 and September 2021. The following key search terms were used*: *“lymphocytes” OR “Neutrophil-to-lymphocyte ratio” AND “Cholangiocarcinoma (CCA)” OR “Biliary tree cancers (BTC)”. Two independent literature searches were carried out by two authors based on the same strategy. Subsequently, no further publications were identified after the reference list and citations search were completed.

### Inclusion and exclusion criteria

Inclusion criteria were:

- Studies investigating the prognostic value of NLR in CCA.
- Reporting of survival data (DFS, OS).

Exclusion criteria were:

- No access to the full text.
- Reviews, case reports, comments or editorials.
- Non-English papers.

### Statistical analysis

The statistical analysis was conducted as previously described^7^. Hazard ratios (HR) and 95% CIs were used to assess the association between NLR and outcomes. Kaplan–Meier curves in combination with Engauge Digitizer version 12.1 were used to extract these information if not directly reported as described previously^14^. RevMan version 5.4 and R project version 4.1.2 were used to analyze and visualize the results. Measures of statistical heterogeneity between studies were calculated (tau, Q, I value) and assessed using the Chi-squared test and assumed to be significant when I^2^ > 50% and/or p < 0.05. A random-effect model and subgroup analysis were preferred when heterogeneity existed, while a fixed-effect model was used when no variance was detected in the data set. Subgroup analysis was carried out to investigate heterogeneity in the studies, while sensitivity analyses were performed to determine the stability of the overall effects. Here, one study at a time was excluded to ensure that no single study would dominate and would be solely responsible for a significant result. Baujat plots were used to investigate the contribution of studies to the heterogeneity as well as pooled outcome and funnel plots were utilized to evaluate publication bias^15^.

### Quality assessment of selected studies

The quality of the included studies was structurally evaluated by 2 reviewers (DL and JB) using the Newcastle–Ottawa scale^16^. The Newcastle–Ottawa scale is composed of the following three quality indicators: outcome assessment, comparability, and selection. Each paper was scored from 0 to 9 based on these parameters.

## Results

### Literature search

**Figure 1:**
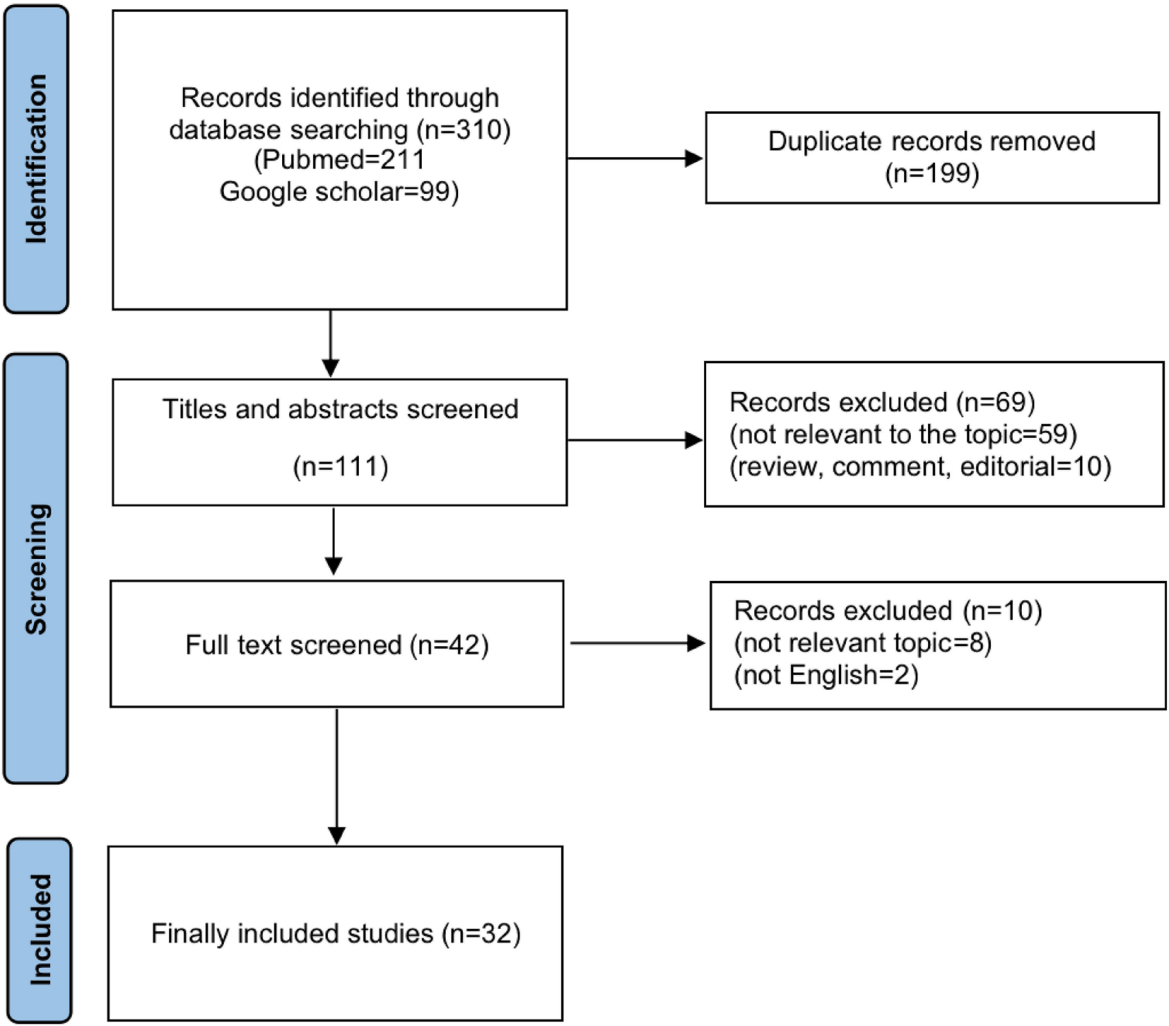
Flowchart of study selection for this study.

The process of selecting publications is depicted in Fig. 1. Initially, 310 articles were identified searching two databases. Subsequently, 199 duplicate records were detected and eliminated. The remaining 111 studies were further assessed for eligibility after titles and abstracts were reviewed and subsequently, a full-text screening was conducted for 42 publications of which finally 32 studies were eligible to be included in this meta-analysis ^17–48^.

### Study characteristics and quality assessment

The key characteristics of the 32 publications analyzed in this manuscript are depicted in Table 1. All publications included were retrospective cohort studies comprising a total of 8572 patients, 6427 of whom had liver resection and 2145 of whom had undergone non-surgical therapy. The mean age of the study populations was 56 to 70 years with males accounting for 31% to 79% of the patients in the investigated data set. NLR cut-off values were different between the studies and obtained using various approaches. Regarding the investigated entities, 19 studies focused on ICCA, 8 on ECCA and 5 studies analyzed both ICCA and ECCA. While all studies reported a correlation between OS and NLR, only 14 reported a correlation between DFS and NLR.

**Table 1:** Characteristics of included studies.

	Author	Year published	Country	Tumor type	Sample size	Stage	Age (median)	Male (%)	Treatment	Follow-up (months, median)	Endpoint	Cut-off value (high expression)
^17^	Zhao JP	2021	China	ICCA	468	NR	58	60.30%	Surgery	NR	OS	NLR ≥ 3
^18^	Ma B	2021	China	ICCA	174	I–IV	58	55.90%	Surgery	25.1	OS/DFS	NLR ≥ 3
^19^	Zhang ZY	2020	China	ICCA	128	I–III	56	55.00%	Surgery	NR	OS/DFS	NLR ≥ 3
^20^	Tsilimigras DI	2020	USA	ICCA	688	I–III	57	60.50%	Surgery	22.3	OS	NLR ≥ 5
^21^	Ohira M	2020	Japan	ICCA	52	I–IV	58	78.84%	Surgery	NR	OS	NLR ≥ 1.93
^22^	Ji F	2020	China	ECCA	59	I–IV	57	55.93%	Surgery	NR	OS	NLR ≥ 2.93
^23^	Huh G	2020	Korea	ICCA	137	III–IV	64	60.60%	Non-surgery	9.9	OS/DFS	NLR ≥ 5
^24^	Filippi L	2020	Latina	ICCA	20	NR	65	45.00%	Non-surgery	12.5	OS	NLR ≥ 2.7
^25^	Zhang Y	2019	China	ICCA	322	I–IV	57	60.25%	Surgery	44	OS/DFS	NLR ≥ 3
^26^	Wu YH	2019	China	ICCA	123	I–IV	57	54.47%	Surgery	29.1	OS	NLR ≥ 2.05
^27^	Sellers CM	2019	USA	ICCA	131	I–IV	65	51.90%	Surgery	13	OS	NLR ≥ 3.96
^28^	Lin J	2019	China	ICCA	218	I–IV	60	56.90%	Surgery	NR	OS	NLR ≥ 2.94
^29^	Hu HJ	2019	China	ECCA	134	I–IV	60	63.01%	Surgery	NR	OS	NLR ≥ 3
^30^	Hoshimoto S	2019	Japan	ECCA	53	I–IV	70	58.00%	Surgery	18	OS/DFS	NLR ≥ 1.97
^31^	Buettner S	2018	Netherlands	ICCA	991	I–IV	59	54.10%	Surgery	29	OS	NLR ≥ 5
^32^	Yoh T	2017	Japan	ICCA	141	I–IV	65	63.00%	Surgery	NR	OS	NLR ≥ 5
^33^	Omichi K	2017	USA	ICCA	119	I–IV	58	57.14%	Non-surgery	NR	OS/DFS	NLR ≥ 3
^34^	Nam K	2017	Korea	ICCA	377	I–IV	60	69.00%	Surgery	NR	OS	NLR ≥ 2.7
^35^	Kitano Y	2017	Japan	ECCA	120	I–IV	58	68.33%	Surgery	NR	OS/DFS	NLR ≥ 2.8
^36^	Cho H	2017	Korea	ICCA	305	III–IV	59	61.50%	Non-surgery	25	OS/DFS	NLR ≥ 2.8
^37^	Okuno M	2016	Japan	ECCA	219	III–IV	65	58.45%	Non-surgery	80.4	OS	NLR ≥ 5
^38^	Okuno M	2016	Japan	ECCA	534	I–IV	66	62.92%	Surgery	78	OS	NLR ≥ 3
^39^	Lin GH	2016	China	ICCA	102	I–IV	58	64.71%	Surgery	NR	OS/DFS	NLR ≥ 3
^40^	Lee BS	2016	Korea	CCA	221	III–IV	62	69.20%	Non-surgery	NR	OS/DFS	NLR ≥ 5
^41^	Ha H	2016	Korea	CCA	534	III–IV	60	65.20%	Non-surgery	95.3	OS	NLR ≥ 3.49
^42^	Beal EW	2016	USA	ECCA	525	I–IV	68	50.67%	Surgery	NR	OS/DFS	NLR ≥ 5
^43^	Chen Q	2016	China	ICCA	322	I–IV	58	60.25%	Surgery	NR	OS/DFS	NLR ≥ 2.49
^44^	Chen Q	2015	China	ICCA	322	I–IV	58	60.25%	Surgery	NR	OS/DFS	NR
^45^	McNamara MG	2014	Canada	CCA	864	I–IV	65	51.39%	Mix*	14.4	OS	NLR ≥ 3
^46^	Iwaku A	2014	USA	CCA	52	III–IV	70	59.62%	Non-surgery	4	OS	NLR ≥ 4
^47^	Dumitrascu T	2013	Romania	ECCA	90	I–IV	58	No	Surgery	68	OS/DFS	NLR ≥ 3.3
^48^	Gomez D	2008	UK	ICCA	27	I–IV	57	31.00%	Surgery	23	OS/DFS	NLR ≥ 5

Mix*, including 326 surgical and 538 non-surgery cases, *CCA* cholangiocarcinoma, *DFS* disease-free surviva, *ECCA* extrahepatic cholangiocarcinoma, *ICCA* intrahepatic cholangiocarcinoma, *NLR* neutrophile-to-lymphocyte ratio, *NR* not reported, *OS* overall survival, *Ref* reference.

The study quality was evaluated between six and nine points on the Newcastle–Ottawa quality assessment scale indicating that the methodology of the investigations was of generally good quality (Table 2).

**Table 2:** Qualities of cohort studies are evaluated by modified Newcastle–Ottawa scale.

Ref	Author	Selection	Comparability	Outcomes	Quality score
^17^	Zhao JP	★★★★	★★	★★	9
^18^	Ma B	★★★	★★	★★	8
^19^	Zhang ZY	★★★★	★★	★★	9
^20^	Tsilimigras DI	★★★★	★★	★★	9
^21^	Ohira M	★★★★	★★	★★	9
^22^	Ji F	★★★★	★★	★★	9
^23^	Huh G	★★★★	★★	★★	9
^24^	Filippi L	★★★★	★★	★★	9
^25^	Zhang Y	★★★★	★★	★★	9
^26^	Wu YH	★★★	★★	★★	8
^27^	Sellers CM	★★★★	★★	★★	9
^28^	Lin J	★★★★	★★	★★	9
^29^	Hu HJ	★★★	★★	★★	8
^30^	Hoshimoto S	★★★	★★	★★	8
^31^	Buettner S	★★★★	★★	★	8
^32^	Yoh T	★★★	★★	★	6
^33^	Omichi K	★★★★	★★	★★	9
^34^	Nam K	★★★★	★★	★	8
^35^	Kitano Y	★★★★	★★	★★	9
^36^	Cho H	★★★★	★★	★	8
^37^	Okuno M	★★★★	★★	★★	9
^38^	Okuno M	★★★★	★★	★★	9
^39^	Lin GH	★★★★	★★	★	8
^40^	Lee BS	★★★★	★★	★★	9
^41^	Ha H	★★★★	★★	★★	9
^42^	Beal EW	★★★★	★★	★★	9
^43^	Chen Q	★★★★	★★	★★	9
^44^	Chen Q	★★★★	★★	★	8
^45^	McNamara MG	★★★★	★★	★★	9
^46^	Iwaku A	★★★★	★★	★★	9
^47^	Dumitrascu T	★★★★	★★	★	8
^48^	Gomez D	★★★★	★★	★	8

The quality of the included studies was assessed under six items of Hayden et al. All included translational studies reporting oncological outcome were evaluated in accordance with the Newcastle–Ottawa scale. The maximum score of the scale is nine points with studies being categorized as low (0–3 points), moderate (4–6 points) and high quality (7–9 points), respectively.

### Correlation between NLR and OS in CCA

Among the 32 publications related to OS, 19 original papers reported that NLR was an independent predictor for impaired OS^18,20,23–27,32–34,38–40^.

**Figure 2:**
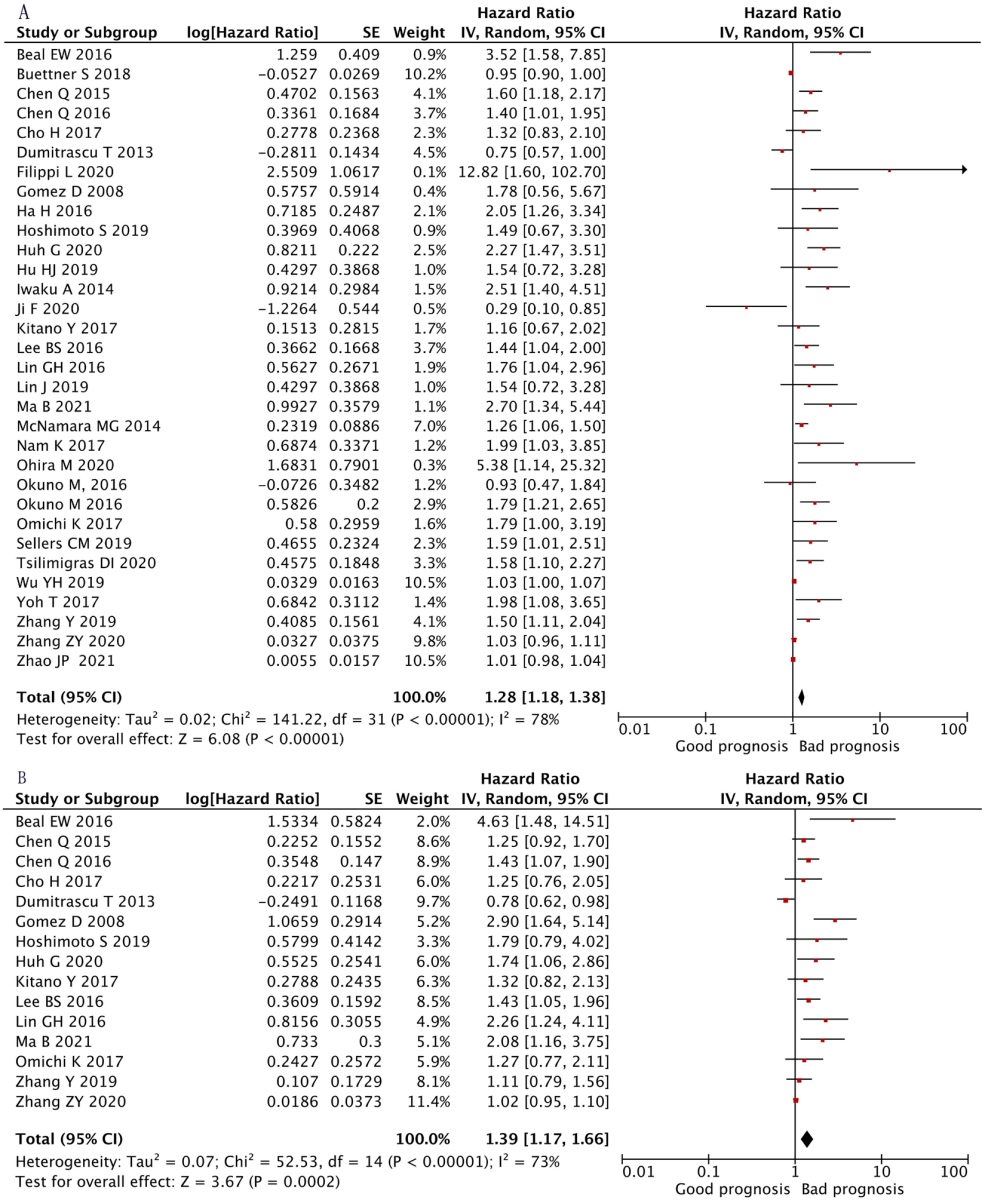
Forest plot of the correlation between NLR and survival in CCA. High NLR values indicated a worse OS (**A**) (HR 1.28, 95% CI 1.18–1.38, p < 0.01) with high heterogeneity (Tau^2^ = 0.02, Chi^2^ = 141.22 p < 0.01, I^2^ = 78%) and a higher NLR level was associated with worse DFS (**B**) (HR  1.39, 95% CI 1.17–1.66, p < 0.01) with high heterogeneity (Tau^2^ = 0.07, Chi^2^ = 52.53 p < 0.01, I^2^ = 73%).

^41–46^, while 12 studies observed no association^17,19,21,28–31,35–37,47,48^ and one study identified NLR as prognostic for a longer OS^22^. The combined analysis of all 32 publications displayed that high NLR values indicated an impaired OS (HR 1.28, 95% CI 1.18–1.38, p < 0.01) with high heterogeneity (I^2^ = 78%, p < 0.01, Fig. 2A).

### Correlation between NLR and DFS in CCA

Among the 15 publications which reported DFS, 7 cohort studies showed that NLR was an independent predictor of reduced DFS in patients with CCA^18,23,39,40,42,43,48^, whereas 7 publications observed no significant relationship between NLR^19,25,30,33,35,36,44^ and DFS and one study showed NLR was a prognostic for a longer DFS^47^. The pooled analysis of all studies showed that a higher NLR level was associated with impaired DFS (HR 1.39, 95% CI 1.17–1.66, p < 0.01) with high heterogeneity (I^2^ = 73%, p = 0.02, Fig. 2B).

### Subgroup analyses of correlation between NLR and survival in CCA

Significant heterogeneity was detected in the HRs of OS for NLR (I^2^ = 78%, p < 0.01, Fig. 2A) and of DFS for NLR (I^2^ = 73%, p < 0.01, Fig. 2B). Thus, causes of the heterogeneity were investigated by subgroup analyses focusing on NLR cut-off values, treatment (surgical vs. non-surgical), cancer type, geographical region, age, size and sample.

For OS, NLR was prognostic in each defined subgroup of treatment type (surgical: p < 0.01; non-surgical: p < 0.01), cut-off value (> 3: p < 0.01; ≤ 3: p < 0.01), geographical region (western: p = 0.02; eastern: p < 0.01), sample size (n ≥ 200: p < 0.01; n < 200: p < 0.01) and age (≥ 60: p < 0.01; < 60: p = 0.01; Table 3, Supplementary Fig. S1) However, in the stratified analysis for cancer type, the pooled analysis of studies exclusively reporting on ECCA showed no significant effect of NLR on OS (p = 0.38), while statistical significance was obtained for ICCA (p < 0.01) and CCA (p < 0.01; Table 3).

**Table 3:** Summary of the subgroup analyses of the correlation between NLR and overall survival in CCA patients.

Subgroup	Number of studies	HR [95%CI]	P value	Heterogeneity	
				I^2^	p
**Cancer type**					
CCA*	4	1.60 [1.20–2.12]	< 0.01	61%	0.05
ICCA	20	1.21 [1.11–1.31]	< 0.01	78%	< 0.01
ECCA	8	1.20 [0.79–1.82]	0.38	75%	< 0.01
**Treatment**					
Surgery	23	1.14 [1.06–1.23]	< 0.01	73%	< 0.01
Non-surgery	9	1.71 [1.39–2.10]	< 0.01	49%	0.05
**Cut-off value**					
NLR > 3	15	1.40 [1.23–1.60]	< 0.01	85%	< 0.01
NLR ≤ 3	17	1.25 [1.10–1.42]	< 0.01	67%	< 0.01
**Region**					
Eastern	21	1.28 [1.17–1.40]	< 0.01	75%	< 0.01
Western	11	1.36 [1.05–1.76]	0.02	83%	< 0.01
**Sample size**					
≥ 200	14	1.29 [1.15–1.45]	< 0.01	80%	< 0.01
< 200	18	1.39 [1.19–1.61]	< 0.01	77%	< 0.01
Age**					
≥ 60	15	1.72 [1.44–2.05]	< 0.01	42%	0.04
< 60	17	1.09 [1.02–1.18]	0.01	40%	< 0.01

*Includes both ICCA and ECCA. **Mean/median age of the study cohort. *ECCA* extrahepatic cholangiocarcinoma, *HR* hazard ratio, *ICCA* intrahepatic cholangiocarcinoma, *NLR* neutrophil-to-lymphocyte ratio.

For DFS, NLR was prognostic in each defined subgroup of treatment type (surgical: p < 0.01; non-surgical: p < 0.01), cut-off value (> 3: p = 0.03; ≤ 3: p < 0.01), sample size (n ≥ 200: p < 0.01; n < 200: p < 0.01) and age (≥ 60: p < 0.01; < 60: p = 0.01; Table 4, Supplementary Fig. S1). Similar to OS, the stratified analysis for cancer type, showed no significant effect of NLR on DFS in ECCA (p = 0.67) while again statistical significance was obtained for ICCA (p < 0.01) and CCA (p = 0.02; Table 4, Supplementary Fig. S2). Also, no prognostic effect of NLR on DFS was obtained for western patients (p = 0.16) in this sub analysis (Table 4, Supplementary Fig. S2).

**Table 4:** Summary of the subgroup analyses of the correlation between NLR and DFS in CCA patients.

Subgroup	Number of studies	HR [95%CI]	P value	Heterogeneity	
				I^2^	p
**Cancer type**					
CCA*	1	1.43 [1.05–1.96]	0.02	-	-
ICCA	10	1.44 [1.17–1.77]	< 0.01	73%	< 0.01
ECCA	3	1.11 [0.68–1.83]	0.67	70%	0.03
**Treatment**					
Surgery	11	1.40 [1.13–1.74]	< 0.01	78%	< 0.01
Non-surgery	4	1.42 [1.15–2.75]	< 0.01	0%	0.78
**Cut-off value**					
NLR > 3	6	1.56 [1.04–2.34]	0.03	84%	< 0.01
NLR ≤ 3	9	1.33 [1.10–1.62]	< 0.01	59%	0.01
**Region**					
Eastern	11	1.36 [1.15–1.61]	< 0.01	62%	< 0.01
Western	4	1.72 [0.81–3.63]	0.16	88%	< 0.01
**Sample size**					
≥ 200	6	1.34 [1.13–1.59]	< 0.01	21%	0.28
< 200	9	1.44 [1.11–1.88]	< 0.01	79%	< 0.01
Age**					
≥ 60	4	1.70 [1.23–2.34]	< 0.01	24%	0.26
< 60	11	1.30 [1.08–1.57]	0.01	74%	< 0.01

*Includes both ICCA and ECCA. **Mean/median age of the study cohort. *ECCA* extrahepatic cholangiocarcinoma, *HR* hazard ratio, *ICCA* intrahepatic cholangiocarcinoma, *NLR* neutrophil-to-lymphocyte ratio.

### Sensitivity analyses of correlation between NLR and prognosis of CCA patients

**Figure 3:**
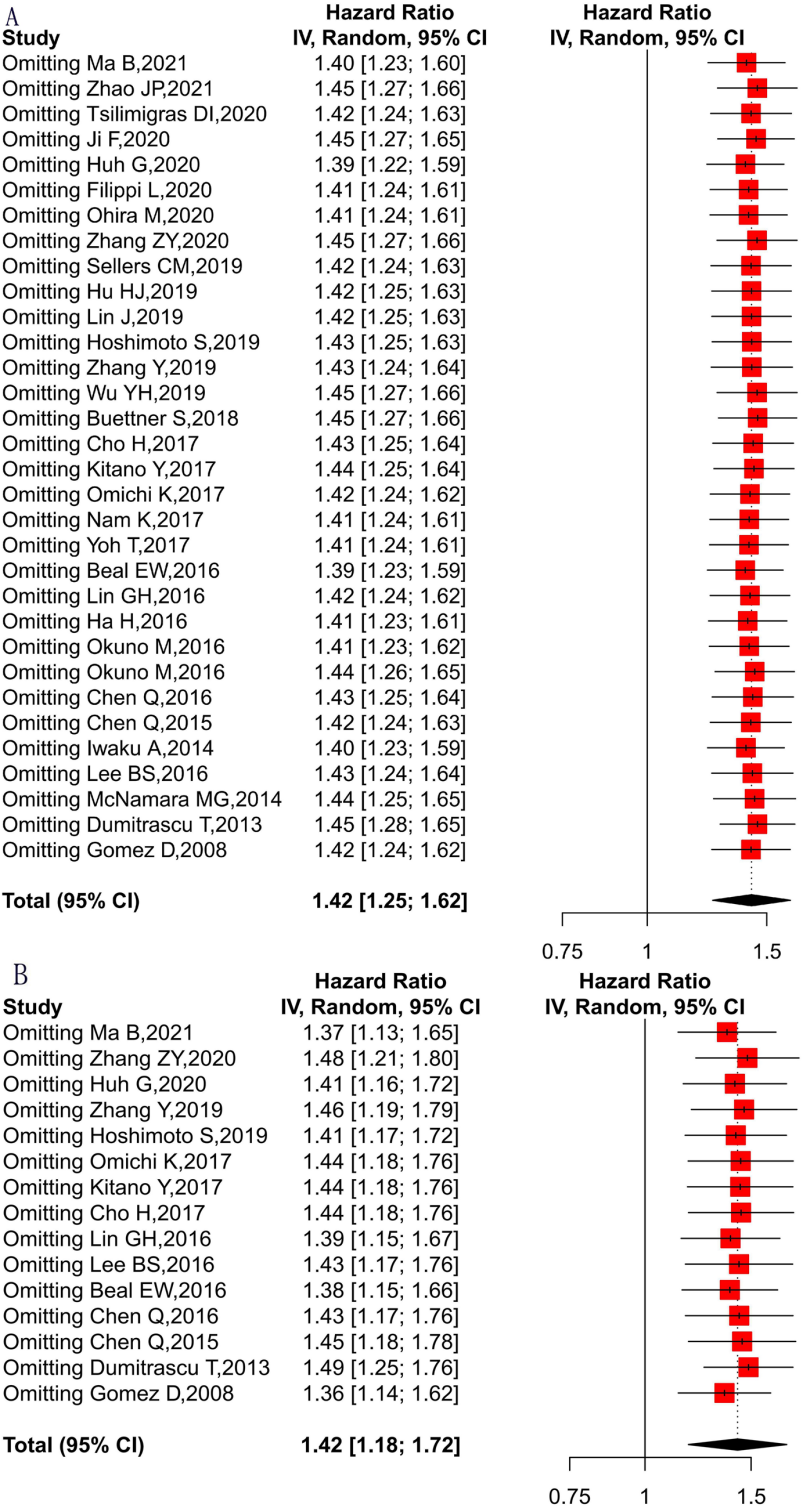
Sensitivity analyses of correlation between NLR and prognosis of CCA patients. Adopting a random effects model in sensitivity analyses, deleting each study in each turn, to further determine the robustness of the prognostic role of NLR. High NLR still displayed an unfavorable effect on OS(A) and DFS(B).

To determine the prognostic robustness of NLR, a random effects model in sensitivity analyses was adopted, deleting each study in each turn. As shown in Fig. 3, the results of the pooled HRs changed in each analysis, but high NLR still displayed an unfavorable effect on OS and DFS. These results indicate that the association between NLR and survival in CCA is certainly robust.

### Contribution of studies to the heterogeneity and pooled outcome

Baujat plots were used to detect studies which overly contributed to the heterogeneity in this meta-analysis. Here, Huh et al. contributed heavily to the overall heterogeneity in this meta-analysis, while Zhao et al. had a significant influence on the overall result and Buettner S et al. influenced both the estimated heterogeneity and the pooled effect (Supplementary Fig. S3A). Similarly, for DFS the study of Ma et al. contributed to the overall heterogeneity of this meta-analysis and Zhang et al. had the greatest impact on the pooled effect (Supplementary Fig. S3B).

### Publication bias

No bias influencing the HRs could be detected as the results from a funnel plot analysis displayed no asymmetry (Supplementary Fig. S4).

## Discussion

Recently, a number of studies have investigated the interaction between inflammation and cancer^8,49^. NLR, as an inflammatory index, has been shown to be associated with various clinical endpoints including long-term prognosis, disease recurrence and response to treatment^50^. Previous meta-analyses demonstrated that high NLR values are linked to poor oncological survival in hepatocellular^51^, breast^52^, esophageal^53^, colorectal^54^, lung^55^, pancreatic cancer^56^. In contrast, a meta-analysis by Templeton et al. identified significant differences in pooled effect estimates when stratifying studies by cancer type and metastatic versus non-metastatic disease, suggesting the prognostic potential of the NLR may not be equal among all patient and cancer subgroups^8^. Considering the effects in other tumor entities, the main research question of the present meta-analysis was whether NLR has also prognostic value in cholangiocarcinoma patients (CCA). To investigate this, 32 studies with a total of 8572 patients were assessed. Results show that high NLR is associated with significantly poor OS and DFS with notable hazard ratios.

Notably, the subgroup analysis revealed that the unfavorable effect of NLR is independent from sample size, age, NLR cut-off value, different treatment types including palliative or curative therapy and geographical region. Although, we observed no statistical significance in DFS for Western patients, the number of included studies from Western countries was limited (n = 4) and displayed a high level of heterogeneity (I^2^ = 88%, p < 0.01, Table 4, Fig. S2). The observation that the prognostic ability of NLR is independent from the applied oncological treatment is of particular interest since the oncological outcome of curative treatment is significantly different to the palliative setting in CCA. While 5-year survival rates higher than 50% have been demonstrated in selected subgroups of CCA patients undergoing curative-intent liver resection, the median OS in the palliative setting remains discouraging (< 12 months) due to lack of effective systemic therapy^57–59^. Given this observation, one might speculate that NLR could be closely associated with the individual tumor biology and therefore might be a suitable prognostic biomarker irrespective of the applied treatment across the oncological stages. Interestingly, Zheng et al. carried out a comparable meta-analysis for hepatocellular carcinoma and also noticed that high NLR is an independent risk factor for DFS and OS in HCC patients in a palliative and curative setting^51^.

Further, the subgroup analysis marginally failed to detect a statistically significant association between oncological outcomes and NLR in patients with ECCA (for OS: HR 1.20, 95% CI 0.79–1.82, p = 0.38; for DFS: HR 1.11, 95% CI 0.68–1.83, p = 0.07). However, the number of studies on ECCA was limited (nOS = 6, nDFS = 3). Furthermore, these reports included the distal as well as perihilar subtype of CCA, resulting in a significant heterogeneity (I^2^ = 60% and 70%, respectively) among the analyzed publications. In addition, ECCA is usually characterized by recurrent cholangitis with following septic complications interfering with long-term survival^5^. As the data suggests a primary association of NLR with OS and DFS, it seems plausible that the prognostic value of NLR might be mitigated in the scenario of ECCA as septic events might also result in deaths which are pre see not cancer-related.

Chronic inflammation is believed to play a notable role in 15% of cancer cases globally and it is accepted that a systemic inflammatory activation is an important player in carcinogenesis and progression^60^. However, the underlying mechanisms explaining how NLR influences oncological outcomes are yet to be explored^7^. In the clinical setting, systemic inflammation is primarily reflected and quantified by changes in blood parameters and can be determined by the counts of various cell components of the peripheral blood (neutrophils, lymphocytes, monocytes and platelets) using standard thresholds^61^. Previous studies have already shown that the association between neutrophils and circulating tumor cells (CTCs) drives cell cycle progression to confer proliferative advantage of CTC clusters, leading to faster metastasis development and enhanced metastatic potential of CTC clusters^62^.

Local and systemic inflammation is often involved in the initial carcinogenesis, cell proliferation, angiogenesis, and metastasis or progression of malignant tumors^6^. Quail et al. have linked neutrophilia to tumor-derived granulocyte colony-stimulating factor (GCSF) which at the same time accelerates tumor development^63^. Other studies depicted that neutrophils itself promote the survival and proliferation of malignant cells by secreting pro-inflammation mediators, such as tumor necrosis factor alpha (TNFa), interleukin (IL) 1, IL 6 and vascular endothelial growth factor^64^. Furthermore, a meta-analysis on NLR in solid tumors in general also demonstrated that high NLR is associated with poor survival in many malignancies, showing a particularly pronounced effect in metastatic advanced disease^8^. Park et al. also found that an elevated NLR is associated with a poor lymphocyte-mediated cytotoxicity against tumor cells characterized by a lower density of tumor-infiltrating lymphocytes (CD3+ and CD8+ T cells) in individuals with colorectal cancer^65^.

Escape from immune surveillance is considered to be a key characteristic of tumorigenesis and cancer progression. Novel treatment modalities, e.g. immune checkpoint inhibitors (ICIs), tumor vaccines and chimeric antigen receptor (CAR) T-cells are currently under investigation and suggested to have high potential to improve treatment^66^. However, in contrast to other solid tumors, the response rates to immunotherapy have not shown satisfying results which may be attributed to the spatial heterogeneity in CCA per se. In fact, there is a lack of reliable prognostic biomarkers and risk-assessment tools which would be suitable to predict the future response to these therapies. This is also considered a main obstacle in the use of immunotherapies in CCA patients. Katayama et al. studied NLR in 81 patients diagnosed with non-small cell lung cancer (NSCLC) who received atezolizumab as monotherapy and observed that patients with high NLR at baseline exhibited shorter progression-free survival and OS compared to those with a low NLR^67^. Li et al. reported that patients receiving ICIs for metastatic disease with NLR < 5 showed significantly longer OS^68^. In addition, Ota et al. studied the data of 98 patients who received nivolumab and found that poor prognostic factors of OS were pretreatment NLR of > 3 and NLR difference of > 2 over 60 days before and after receiving nivolumab. Those individuals with NLR difference > 2 displayed a longer median OS^69^. Hence, NLR holds promise to predict treatment response to ICIs in CCA as well.

The pure prognostic value of NLR was frequently investigated in other tumor entities. Yang et al*.* conducted a meta-analysis based 1804 pancreatic cancer patients and showed that high NLR was linked to reduced OS in individuals treated by chemotherapy or surgical resection. Furthermore, a high NLR was associated to tumor metastasis, poor tumor differentiation, poor performance status and elevated carbohydrate antigen 19–9^56^. Moreover, NLR indicated reduced OS and DFS in breast cancer patients, with its prognostic value being retained across different clinicopathologic parameters such disease stage and subtypes^70^. In patients with esophageal cancer, a higher pretreatment NLR was linked to shorter survival as well as deeper tumor invasion and the presence of lymph node metastases^53^. Surprisingly, ethnicity had also an impact on certain studies. For example, Gu et al*.* discovered that NLR has consistent prognostic value in metastatic castration-resistant prostate cancer patients and predicts poor PFS/RFS in Asian, but not in Caucasian individuals^71^. In colon cancer patients undergoing a variety of treatments such as resection of the primary tumor, palliative chemotherapy and resection or ablation for liver metastases, higher preoperative NLR was prognostic for a lower survival rate^72^. Given these reports in other solid tumors, our results were in line with previous results and support a general role NLR as prognostic in solid malignancies.

As all meta-analyses with limited available studies, the analysis has certainly limitations due to the lack of high-level evidence:

- The study comprised a variety of methodologies and, most importantly, different NLR cutoff levels.
- Further, the included studies were retrospective in nature and therefore have an inherent potential of selection bias. As several studies did not explicitly report HRs and CIs, these variables were extrapolated from the Kaplan–Meier curves in some papers^27,28,36,38,46,48^.
- A detailed investigation of the association between NLR and tumor clinicopathological characteristics was not feasible as the published data were unfortunately not detailed enough. This also accounts for the different types of cholangiocarcinoma as some studies include both ICCA and ECCA in a unified analysis as well as the distinct molecular subtypes of CCA.

Future research should therefore focus on the role of NLR in different subtypes and the identification of a uniform NLR cut-off to facilitate the implantation into clinical management of patients. Despite these obvious limitations, the present meta-analysis has also inherent strengths:

- Representative data set especially for patients undergoing surgical treatment.
- Detailed sub-analysis for study sample size, age, NLR cut-off value, geographical region, tumor subtypes and different types.
- The inclusion of a sensitivity analysis indicating that no single study is responsible for the overall significant effect of NLR on OS and DFS.

## Conclusions

Considering the aforementioned limitations and the limited available sample size, this study indicates a notable likelihood of NLR to be prognostic for reduced OS and DFS. Elevated NLR before treatment might therefore serve as biomarker for reduced oncological outcome (OS and DFS) in CCA patients. As patients undergoing surgery for CCA display high rates of perioperative morbidity and mortality, preoperative patient selection is fundamental to balance surgical risk with oncological benefits. Here, NLR provides additional information for treatment selection and risk stratification.

## Supplementary Information

Supplementary Information.

## Data Availability

Available upon request. JB and UPN had full access to the data and act both as guarantor for the data.

## Abbreviations

BTC: Biliary tree cancers
CAR: Chimeric antigen receptor
CCA: Cholangiocarcinoma
CI: Confidence interval
CRP: C-reactive protein
CTCs: Circulating tumor cells
DCCA: Distal cholangiocarcinoma
DFS: Disease-free survival
ECCA: Extrahepatic cholangiocarcinoma
GCSF: Granulocyte colony-stimulating factor
GPS: Glasgow prognostic score
HR: Hazard ratio
ICC: Intrahepatic cholangiocarcinoma
ICIs: Immune checkpoint inhibitors
GCSF: Granulocyte colony-stimulating factor
NLR: Neutrophil-to-lymphocyte ratio
NR: No reported
NSCLC: Non-small cell lung cancer
OS: Overall survival
PCCA: Perihilar cholangiocarcinoma
PROSPERO: Prospective register of systematic reviews
Ref: Reference
