# Supplementary material for: The prognostic value of neutrophil-to-lymphocyte ratio in cholangiocarcinoma: a systematic review and meta-analysis
Source: Sci Rep. 2022 Jul 25;12:12691. doi: 10.1038/s41598-022-16727-w (PMC9314341; doi:10.1038/s41598-022-16727-w)

Figure S1 Stratified Forest plots of the association between the NLR and OS

A

Study or Subgroup	log[Hazard Ratio]	SE	Weight	Hazard Ratio IV, Random, 95% CI
**1.3.1 CCA**				
Ha H 2016	0.7185	0.2487	19.1%	2.05 [1.26, 3.34]
Iwaku A 2014	0.9214	0.2984	15.3%	2.51 [1.40, 4.51]
Lee BS 2016	0.3662	0.1668	27.8%	1.44 [1.04, 2.00]
McNamara MG 2014	0.2319	0.0886	37.7%	1.26 [1.06, 1.50]
**Subtotal (95% CI)**			**100.0%**	**1.60 [1.20, 2.12]**
Heterogeneity: $Tau^2 = 0.05$; $Chi^2 = 7.63$, df = 3 (P = 0.05); $I^2 = 61\%$				
Test for overall effect: Z = 3.23 (P = 0.001)				
**1.3.2 ICCA**				
Buettner S 2018	−0.0527	0.0269	16.0%	0.95 [0.90, 1.00]
Chen Q 2015	0.4702	0.1563	4.9%	1.60 [1.18, 2.17]
Chen Q 2016	0.3361	0.1684	4.4%	1.40 [1.01, 1.95]
Cho H 2017	0.2778	0.2368	2.5%	1.32 [0.83, 2.10]
Filippi L 2020	2.5509	1.0617	0.1%	12.82 [1.60, 102.70]
Gomez D 2008	0.5757	0.5914	0.5%	1.78 [0.56, 5.67]
Huh G 2020	0.8211	0.222	2.8%	2.27 [1.47, 3.51]
Lin GH 2016	0.5627	0.2671	2.0%	1.76 [1.04, 2.96]
Lin J 2019	0.4297	0.3868	1.0%	1.54 [0.72, 3.28]
Ma B 2021	0.9927	0.3579	1.2%	2.70 [1.34, 5.44]
Nam K 2017	0.6874	0.3371	1.3%	1.99 [1.03, 3.85]
Ohira M 2020	1.6831	0.7901	0.3%	5.38 [1.14, 25.32]
Omichi K 2017	0.58	0.2959	1.7%	1.79 [1.00, 3.19]
Sellers CM 2019	0.4655	0.2324	2.6%	1.59 [1.01, 2.51]
Tsilimigras DI 2020	0.4575	0.1848	3.8%	1.58 [1.10, 2.27]
Wu YH 2019	0.0329	0.0163	16.7%	1.03 [1.00, 1.07]
Yoh T 2017	0.6842	0.3112	1.6%	1.98 [1.08, 3.65]
Zhang Y 2019	0.4085	0.1561	4.9%	1.50 [1.11, 2.04]
Zhang ZY 2020	0.0327	0.0375	15.0%	1.03 [0.96, 1.11]
Zhao JP 2021	0.0055	0.0157	16.7%	1.01 [0.98, 1.04]
**Subtotal (95% CI)**			**100.0%**	**1.21 [1.11, 1.31]**
Heterogeneity: $Tau^2 = 0.01$; $Chi^2 = 86.00$, df = 19 (P < 0.00001); $I^2 = 78\%$				
Test for overall effect: Z = 4.63 (P < 0.00001)				
**1.3.3 ECCA**				
Beal EW 2016	1.259	0.409	10.9%	3.52 [1.58, 7.85]
Dumitrascu T 2013	−0.2811	0.1434	16.9%	0.75 [0.57, 1.00]
Hoshimoto S 2019	0.3969	0.4068	10.9%	1.49 [0.67, 3.30]
Hu HJ 2019	0.4297	0.3868	11.3%	1.54 [0.72, 3.28]
Ji F 2020	−1.2264	0.544	8.3%	0.29 [0.10, 0.85]
Kitano Y 2017	0.1513	0.2815	13.8%	1.16 [0.67, 2.02]
Okuno M, 2016	−0.0726	0.3482	12.2%	0.93 [0.47, 1.84]
Okuno M 2016	0.5826	0.2	15.7%	1.79 [1.21, 2.65]
**Subtotal (95% CI)**			**100.0%**	**1.20 [0.79, 1.82]**
Heterogeneity: $Tau^2 = 0.24$; $Chi^2 = 28.39$, df = 7 (P = 0.0002); $I^2 = 75\%$				
Test for overall effect: Z = 0.87 (P = 0.38)				

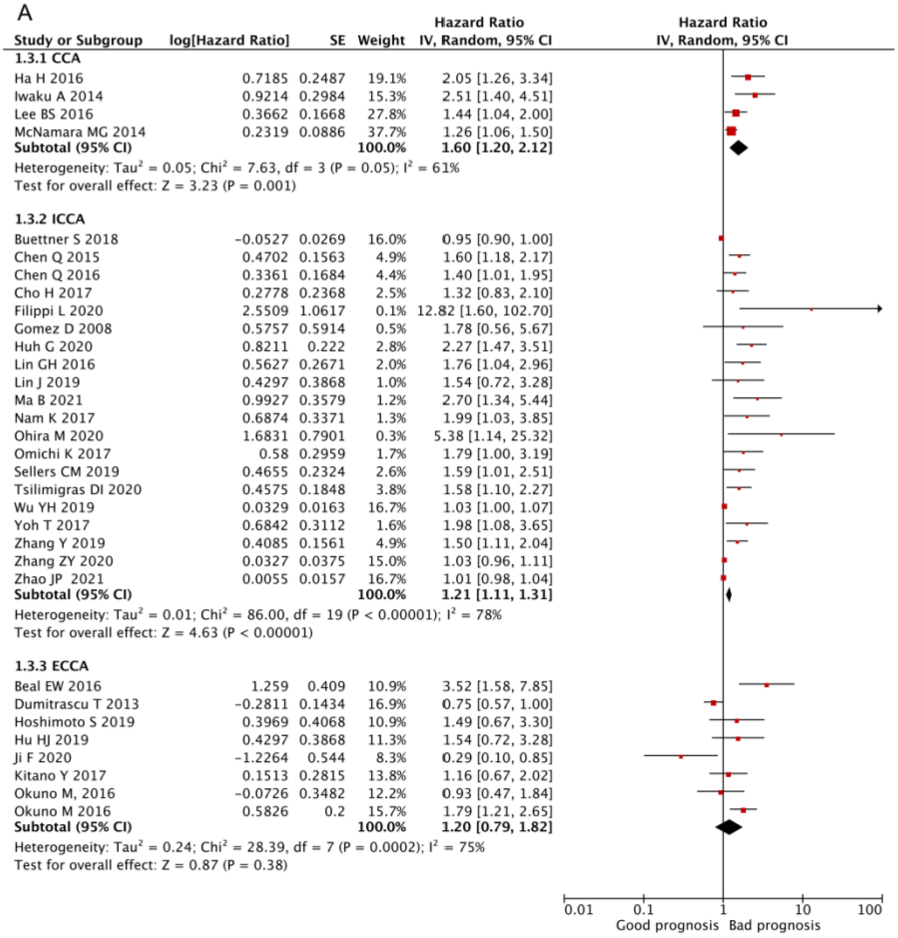

B

Study or Subgroup	log[Hazard Ratio]	SE	Weight	Hazard Ratio IV, Random, 95% CI
**1.2.1 Surgery**				
Beal EW 2016	1.259	0.409	0.8%	3.52 [1.58, 7.85]
Buettner S 2018	−0.0527	0.0269	14.9%	0.95 [0.90, 1.00]
Chen Q 2015	0.4702	0.1563	4.2%	1.60 [1.18, 2.17]
Chen Q 2016	0.3361	0.1684	3.8%	1.40 [1.01, 1.95]
Dumitrascu T 2013	−0.2811	0.1434	4.8%	0.75 [0.57, 1.00]
Gomez D 2008	0.5757	0.5914	0.4%	1.78 [0.56, 5.67]
Hoshimoto S 2019	0.3969	0.4068	0.8%	1.49 [0.67, 3.30]
Hu HJ 2019	0.4297	0.3868	0.9%	1.54 [0.72, 3.28]
Ji F 2020	−1.2264	0.544	0.5%	0.29 [0.10, 0.85]
Kitano Y 2017	0.1513	0.2815	1.6%	1.16 [0.67, 2.02]
Lin GH 2016	0.5627	0.2671	1.8%	1.76 [1.04, 2.96]
Lin J 2019	0.4297	0.3868	0.9%	1.54 [0.72, 3.28]
Ma B 2021	0.9927	0.3579	1.0%	2.70 [1.34, 5.44]
McNamara MG 2014	0.143	0.144	4.8%	1.15 [0.87, 1.53]
Nam K 2017	0.6874	0.3371	1.1%	1.99 [1.03, 3.85]
Ohira M 2020	1.6831	0.7901	0.2%	5.38 [1.14, 25.32]
Okuno M, 2016	−0.0726	0.3482	1.1%	0.93 [0.47, 1.84]
Sellers CM 2019	0.4655	0.2324	2.2%	1.59 [1.01, 2.51]
Tsilimigras DI 2020	0.4575	0.1848	3.3%	1.58 [1.10, 2.27]
Wu YH 2019	0.0329	0.0163	15.7%	1.03 [1.00, 1.07]
Yoh T 2017	0.6842	0.3112	1.3%	1.98 [1.08, 3.65]
Zhang Y 2019	0.4085	0.1561	4.3%	1.50 [1.11, 2.04]
Zhang ZY 2020	0.0327	0.0375	13.9%	1.03 [0.96, 1.11]
Zhao JP 2021	0.0055	0.0157	15.7%	1.01 [0.98, 1.04]
**Subtotal (95% CI)**			**100.0%**	**1.14 [1.06, 1.23]**
Heterogeneity: $Tau^2 = 0.01$; $Chi^2 = 84.45$, df = 23 ($P < 0.00001$); $I^2 = 73\%$				
Test for overall effect: Z = 3.51 (P = 0.0005)				
**1.2.2 Non-Surgery**				
Cho H 2017	0.2778	0.2368	11.1%	1.32 [0.83, 2.10]
Filippi L 2020	2.5509	1.0617	1.0%	12.82 [1.60, 102.70]
Ha H 2016	0.7185	0.2487	10.5%	2.05 [1.26, 3.34]
Huh G 2020	0.8211	0.222	11.9%	2.27 [1.47, 3.51]
Iwaku A 2014	0.9214	0.2984	8.4%	2.51 [1.40, 4.51]
Lee BS 2016	0.3662	0.1668	15.5%	1.44 [1.04, 2.00]
McNamara MG 2014	0.2322	0.1084	19.9%	1.26 [1.02, 1.56]
Okuno M 2016	0.5826	0.2	13.2%	1.79 [1.21, 2.65]
Omichi K 2017	0.58	0.2959	8.5%	1.79 [1.00, 3.19]
**Subtotal (95% CI)**			**100.0%**	**1.71 [1.39, 2.10]**
Heterogeneity: $Tau^2 = 0.04$; $Chi^2 = 15.82$, df = 8 ($P = 0.05$); $I^2 = 49\%$				
Test for overall effect: Z = 5.07 ($P < 0.00001$)				

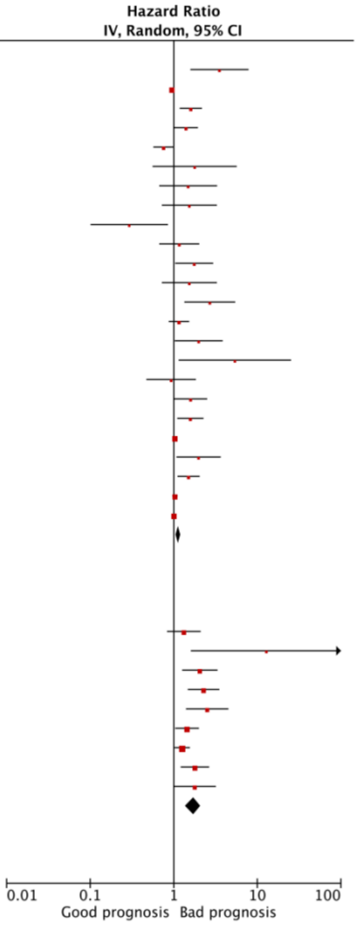

C

Study or Subgroup	log[Hazard Ratio]	SE	Weight	Hazard Ratio IV, Random, 95% CI
**1.5.1 NLR > 3**				
Beal EW 2016	1.259	0.409	2.3%	3.52 [1.58, 7.85]
Buettner S 2018	−0.0527	0.0269	14.0%	0.95 [0.90, 1.00]
Chen Q 2015	0.4702	0.1563	8.0%	1.60 [1.18, 2.17]
Dumitrascu T 2013	−0.2811	0.1434	8.6%	0.75 [0.57, 1.00]
Gomez D 2008	0.5757	0.5914	1.2%	1.78 [0.56, 5.67]
Ha H 2016	0.7185	0.2487	4.8%	2.05 [1.26, 3.34]
Huh G 2020	0.8211	0.222	5.6%	2.27 [1.47, 3.51]
Iwaku A 2014	0.9214	0.2984	3.7%	2.51 [1.40, 4.51]
Lee BS 2016	0.3662	0.1668	7.6%	1.44 [1.04, 2.00]
Okuno M 2016	0.5826	0.2	6.3%	1.79 [1.21, 2.65]
Sellers CM 2019	0.4655	0.2324	5.3%	1.59 [1.01, 2.51]
Tsilimigras DI 2020	0.4575	0.1848	6.9%	1.58 [1.10, 2.27]
Wu YH 2019	0.0329	0.0163	14.2%	1.03 [1.00, 1.07]
Yoh T 2017	0.6842	0.3112	3.5%	1.98 [1.08, 3.65]
Zhang Y 2019	0.4085	0.1561	8.1%	1.50 [1.11, 2.04]
**Subtotal (95% CI)**			**100.0%**	**1.40 [1.23, 1.60]**
Heterogeneity: $Tau^2 = 0.03$; $Chi^2 = 92.11$, df = 14 ($P < 0.00001$); $I^2 = 85\%$				
Test for overall effect: Z = 5.04 ($P < 0.00001$)				
**1.5.2 NLR =<3**				
Chen Q 2016	0.3361	0.1684	8.6%	1.40 [1.01, 1.95]
Cho H 2017	0.2778	0.2368	5.5%	1.32 [0.83, 2.10]
Filippi L 2020	2.5509	1.0617	0.4%	12.82 [1.60, 102.70]
Hoshimoto S 2019	0.3969	0.4068	2.2%	1.49 [0.67, 3.30]
Hu HJ 2019	0.4297	0.3868	2.4%	1.54 [0.72, 3.28]
Ji F 2020	−1.2264	0.544	1.3%	0.29 [0.10, 0.85]
Kitano Y 2017	0.1513	0.2815	4.2%	1.16 [0.67, 2.02]
Lin GH 2016	0.5627	0.2671	4.5%	1.76 [1.04, 2.96]
Lin J 2019	0.4297	0.3868	2.4%	1.54 [0.72, 3.28]
Ma B 2021	0.9927	0.3579	2.8%	2.70 [1.34, 5.44]
McNamara MG 2014	0.2319	0.0886	15.0%	1.26 [1.06, 1.50]
Nam K 2017	0.6874	0.3371	3.1%	1.99 [1.03, 3.85]
Ohira M 2020	1.6831	0.7901	0.6%	5.38 [1.14, 25.32]
Okuno M, 2016	−0.0726	0.3482	2.9%	0.93 [0.47, 1.84]
Omichi K 2017	0.58	0.2959	3.9%	1.79 [1.00, 3.19]
Zhang ZY 2020	0.0327	0.0375	19.5%	1.03 [0.96, 1.11]
Zhao JP 2021	0.0055	0.0157	20.6%	1.01 [0.98, 1.04]
**Subtotal (95% CI)**			**100.0%**	**1.25 [1.10, 1.42]**
Heterogeneity: $Tau^2 = 0.02$; $Chi^2 = 48.99$, df = 16 ($P < 0.0001$); $I^2 = 67\%$				
Test for overall effect: Z = 3.44 (P = 0.0006)				

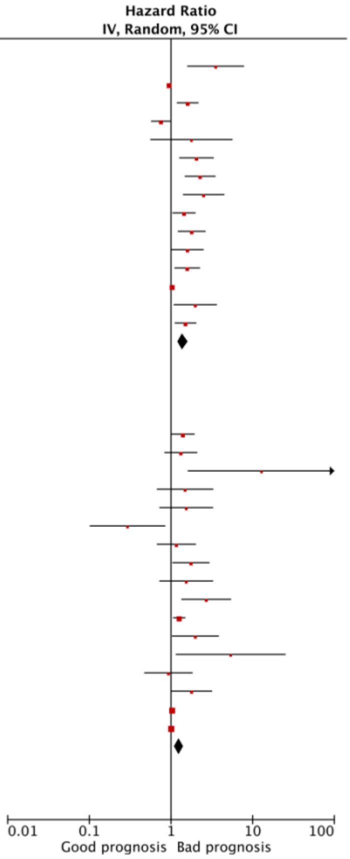

D

Study or Subgroup	log[Hazard Ratio]	SE	Weight	Hazard Ratio IV, Random, 95% CI
**1.4.2 Eastern**				
Chen Q 2015	0.4702	0.1563	5.6%	1.60 [1.18, 2.17]
Chen Q 2016	0.3361	0.1684	5.0%	1.40 [1.01, 1.95]
Cho H 2017	0.2778	0.2368	3.0%	1.32 [0.83, 2.10]
Ha H 2016	0.7185	0.2487	2.7%	2.05 [1.26, 3.34]
Hoshimoto S 2019	0.3969	0.4068	1.1%	1.49 [0.67, 3.30]
Huh G 2020	0.8211	0.222	3.3%	2.27 [1.47, 3.51]
Hu HJ 2019	0.4297	0.3868	1.2%	1.54 [0.72, 3.28]
Kitano Y 2017	0.1513	0.2815	2.2%	1.16 [0.67, 2.02]
Lee BS 2016	0.3662	0.1668	5.1%	1.44 [1.04, 2.00]
Lin GH 2016	0.5627	0.2671	2.4%	1.76 [1.04, 2.96]
Lin J 2019	0.4297	0.3868	1.2%	1.54 [0.72, 3.28]
Ma B 2021	0.9927	0.3579	1.4%	2.70 [1.34, 5.44]
Nam K 2017	0.6874	0.3371	1.6%	1.99 [1.03, 3.85]
Ohira M 2020	1.6831	0.7901	0.3%	5.38 [1.14, 25.32]
Okuno M, 2016	−0.0726	0.3482	1.5%	0.93 [0.47, 1.84]
Okuno M 2016	0.5826	0.2	3.9%	1.79 [1.21, 2.65]
Wu YH 2019	0.0329	0.0163	17.5%	1.03 [1.00, 1.07]
Yoh T 2017	0.6842	0.3112	1.8%	1.98 [1.08, 3.65]
Zhang Y 2019	0.4085	0.1561	5.6%	1.50 [1.11, 2.04]
Zhang ZY 2020	0.0327	0.0375	15.9%	1.03 [0.96, 1.11]
Zhao JP 2021	0.0055	0.0157	17.5%	1.01 [0.98, 1.04]
**Subtotal (95% CI)**			**100.0%**	**1.28 [1.17, 1.40]**
Heterogeneity: $Tau^2 = 0.01$; $Chi^2 = 79.45$, df = 20 (P < 0.00001); $I^2 = 75\%$				
Test for overall effect: Z = 5.57 (P < 0.00001)				
**1.4.3 Western**				
Beal EW 2016	1.259	0.409	6.2%	3.52 [1.58, 7.85]
Buettner S 2018	−0.0527	0.0269	15.9%	0.95 [0.90, 1.00]
Dumitrascu T 2013	−0.2811	0.1434	13.4%	0.75 [0.57, 1.00]
Filippi L 2020	2.5509	1.0617	1.4%	12.82 [1.60, 102.70]
Gomez D 2008	0.5757	0.5914	3.7%	1.78 [0.56, 5.67]
Iwaku A 2014	0.9214	0.2984	8.7%	2.51 [1.40, 4.51]
Ji F 2020	−1.2264	0.544	4.2%	0.29 [0.10, 0.85]
McNamara MG 2014	0.2319	0.0886	14.9%	1.26 [1.06, 1.50]
Omichi K 2017	0.58	0.2959	8.8%	1.79 [1.00, 3.19]
Sellers CM 2019	0.4655	0.2324	10.6%	1.59 [1.01, 2.51]
Tsilimigras DI 2020	0.4575	0.1848	12.1%	1.58 [1.10, 2.27]
**Subtotal (95% CI)**			**100.0%**	**1.36 [1.05, 1.76]**
Heterogeneity: $Tau^2 = 0.11$; $Chi^2 = 59.26$, df = 10 (P < 0.00001); $I^2 = 83\%$				
Test for overall effect: Z = 2.36 (P = 0.02)				

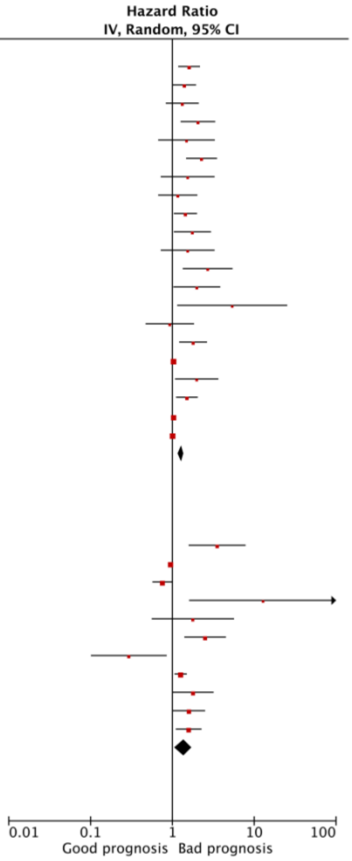

E

Study or Subgroup	log[Hazard Ratio]	SE	Weight	Hazard Ratio IV, Random, 95% CI
**1.6.1 Sample size >=200**				
Beal EW 2016	1.259	0.409	1.8%	3.52 [1.58, 7.85]
Buettner S 2018	−0.0527	0.0269	16.5%	0.95 [0.90, 1.00]
Chen Q 2015	0.4702	0.1563	7.6%	1.60 [1.18, 2.17]
Chen Q 2016	0.3361	0.1684	7.0%	1.40 [1.01, 1.95]
Cho H 2017	0.2778	0.2368	4.4%	1.32 [0.83, 2.10]
Lee BS 2016	0.3662	0.1668	7.1%	1.44 [1.04, 2.00]
Lin J 2019	0.4297	0.3868	2.0%	1.54 [0.72, 3.28]
McNamara MG 2014	0.2319	0.0886	12.2%	1.26 [1.06, 1.50]
Nam K 2017	0.6874	0.3371	2.5%	1.99 [1.03, 3.85]
Okuno M, 2016	−0.0726	0.3482	2.4%	0.93 [0.47, 1.84]
Okuno M 2016	0.5826	0.2	5.6%	1.79 [1.21, 2.65]
Tsilimigras DI 2020	0.4575	0.1848	6.2%	1.58 [1.10, 2.27]
Zhang Y 2019	0.4085	0.1561	7.6%	1.50 [1.11, 2.04]
Zhao JP 2021	0.0055	0.0157	16.9%	1.01 [0.98, 1.04]
**Subtotal (95% CI)**			**100.0%**	**1.29 [1.15, 1.45]**
Heterogeneity: $Tau^2 = 0.02$; $Chi^2 = 65.06$, df = 13 (P < 0.00001); $I^2 = 80\%$				
Test for overall effect: Z = 4.45 (P < 0.00001)				
**1.6.2 Sample size <200**				
Dumitrascu T 2013	−0.2811	0.1434	10.3%	0.75 [0.57, 1.00]
Filippi L 2020	2.5509	1.0617	0.5%	12.82 [1.60, 102.70]
Gomez D 2008	0.5757	0.5914	1.5%	1.78 [0.56, 5.67]
Ha H 2016	0.7185	0.2487	6.0%	2.05 [1.26, 3.34]
Hoshimoto S 2019	0.3969	0.4068	2.9%	1.49 [0.67, 3.30]
Huh G 2020	0.8211	0.222	6.9%	2.27 [1.47, 3.51]
Hu HJ 2019	0.4297	0.3868	3.2%	1.54 [0.72, 3.28]
Iwaku A 2014	0.9214	0.2984	4.7%	2.51 [1.40, 4.51]
Ji F 2020	−1.2264	0.544	1.8%	0.29 [0.10, 0.85]
Kitano Y 2017	0.1513	0.2815	5.1%	1.16 [0.67, 2.02]
Lin GH 2016	0.5627	0.2671	5.5%	1.76 [1.04, 2.96]
Ma B 2021	0.9927	0.3579	3.6%	2.70 [1.34, 5.44]
Ohira M 2020	1.6831	0.7901	0.9%	5.38 [1.14, 25.32]
Omichi K 2017	0.58	0.2959	4.8%	1.79 [1.00, 3.19]
Sellers CM 2019	0.4655	0.2324	6.5%	1.59 [1.01, 2.51]
Wu YH 2019	0.0329	0.0163	15.9%	1.03 [1.00, 1.07]
Yoh T 2017	0.6842	0.3112	4.4%	1.98 [1.08, 3.65]
Zhang ZY 2020	0.0327	0.0375	15.4%	1.03 [0.96, 1.11]
**Subtotal (95% CI)**			**100.0%**	**1.39 [1.19, 1.61]**
Heterogeneity: $Tau^2 = 0.04$; $Chi^2 = 73.54$, df = 17 (P < 0.00001); $I^2 = 77\%$				
Test for overall effect: Z = 4.25 (P < 0.0001)				

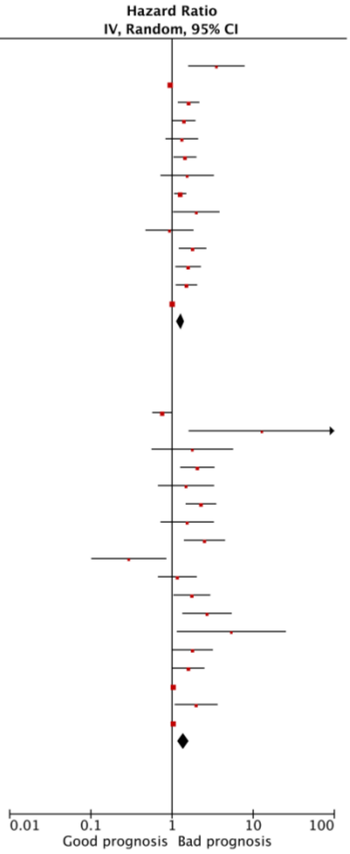

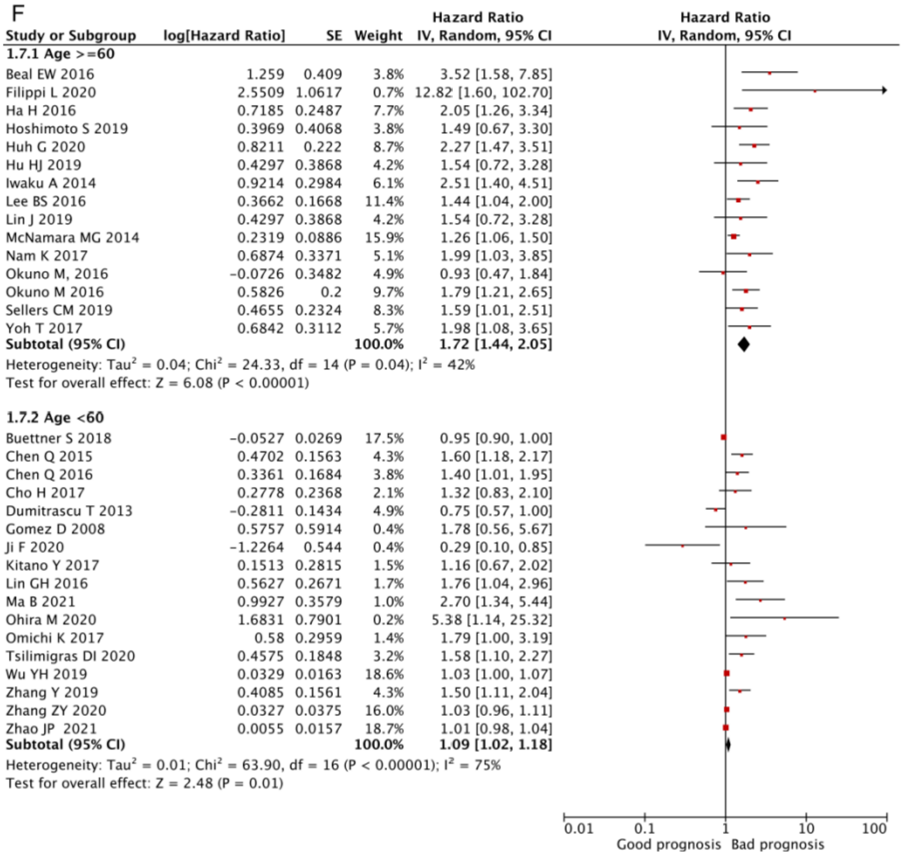

Subgroup analysis of association between the NLR and OS based on cancer type(A), treatment (B), NLR cut-off values(C), region(D), sample size (E) and age(F). A random-effects model was used. CCA, cholangiocarcinoma; ECCA, extrahepatic cholangiocarcinoma; ICCA, intrahepatic cholangiocarcinoma; OS, overall survival; NLR, neutrophil to lymphocytes ratio

Figure S2 Stratified Forest plots of the association between the NLR and DFS

A

Study or Subgroup	log[Hazard Ratio]	SE	Weight	Hazard Ratio IV, Random, 95% CI
**2.3.1 CCA**				
Lee BS 2016	0.3609	0.1592	100.0%	1.43 [1.05, 1.96]
**Subtotal (95% CI)**			**100.0%**	**1.43 [1.05, 1.96]**
Heterogeneity: Not applicable				
Test for overall effect: Z = 2.27 (P = 0.02)				
**2.3.2 ICCA**				
Chen Q 2015	0.2252	0.1552	12.3%	1.25 [0.92, 1.70]
Chen Q 2016	0.3548	0.147	12.6%	1.43 [1.07, 1.90]
Cho H 2017	0.2217	0.2531	8.6%	1.25 [0.76, 2.05]
Gomez D 2008	1.0659	0.2914	7.4%	2.90 [1.64, 5.14]
Huh G 2020	0.5525	0.2541	8.6%	1.74 [1.06, 2.86]
Lin GH 2016	0.8156	0.3055	7.0%	2.26 [1.24, 4.11]
Ma B 2021	0.733	0.3	7.2%	2.08 [1.16, 3.75]
Omichi K 2017	0.2427	0.2572	8.5%	1.27 [0.77, 2.11]
Zhang Y 2019	0.107	0.1729	11.6%	1.11 [0.79, 1.56]
Zhang ZY 2020	0.0186	0.0373	16.3%	1.02 [0.95, 1.10]
**Subtotal (95% CI)**			**100.0%**	**1.44 [1.17, 1.77]**
Heterogeneity: $Tau^2 = 0.07$; $Chi^2 = 33.04$, df = 9 (P = 0.0001); $I^2 = 73\%$				
Test for overall effect: Z = 3.41 (P = 0.0006)				
**2.3.3 ECCA**				
Dumitrascu T 2013	−0.2491	0.1168	44.8%	0.78 [0.62, 0.98]
Hoshimoto S 2019	0.5799	0.4142	21.3%	1.79 [0.79, 4.02]
Kitano Y 2017	0.2788	0.2435	33.9%	1.32 [0.82, 2.13]
**Subtotal (95% CI)**			**100.0%**	**1.11 [0.68, 1.83]**
Heterogeneity: $Tau^2 = 0.13$; $Chi^2 = 6.74$, df = 2 (P = 0.03); $I^2 = 70\%$				
Test for overall effect: Z = 0.42 (P = 0.67)				

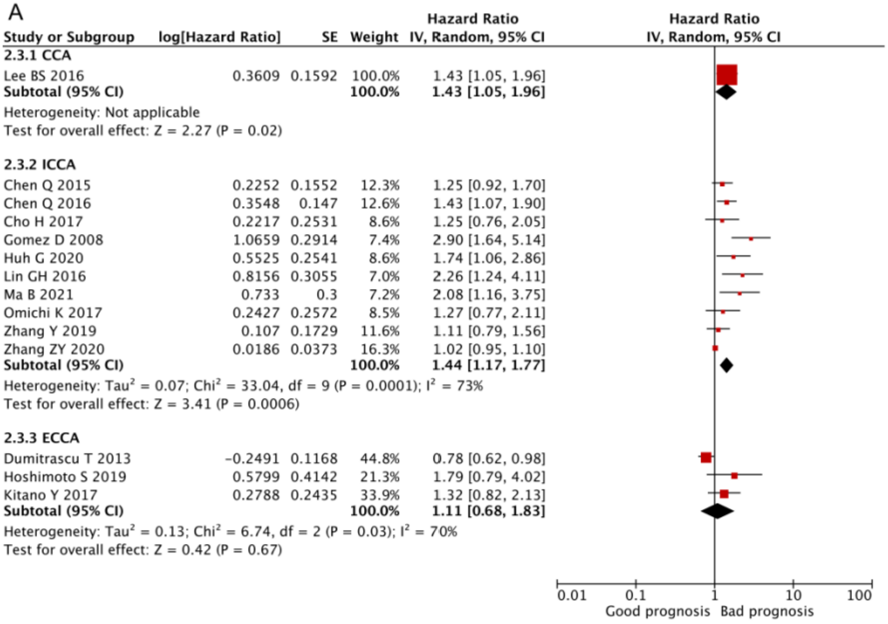

C

Study or Subgroup	log[Hazard Ratio]	SE	Weight	Hazard Ratio IV, Random, 95% CI
**2.5.1 NLR > 3**				
Beal EW 2016	1.5334	0.5824	8.1%	4.63 [1.48, 14.51]
Chen Q 2015	0.2252	0.1552	19.7%	1.25 [0.92, 1.70]
Dumitrascu T 2013	−0.2491	0.1168	20.6%	0.78 [0.62, 0.98]
Gomez D 2008	1.0659	0.2914	15.4%	2.90 [1.64, 5.14]
Huh G 2020	0.5525	0.2541	16.6%	1.74 [1.06, 2.86]
Lee BS 2016	0.3609	0.1592	19.6%	1.43 [1.05, 1.96]
**Subtotal (95% CI)**			**100.0%**	**1.56 [1.04, 2.34]**
Heterogeneity: $Tau^2 = 0.20$; $Chi^2 = 31.64$, df = 5 (P < 0.00001); $I^2 = 84\%$				
Test for overall effect: Z = 2.13 (P = 0.03)				
**2.5.2 NLR =<3**				
Chen Q 2016	0.3548	0.147	15.6%	1.43 [1.07, 1.90]
Cho H 2017	0.2217	0.2531	9.3%	1.25 [0.76, 2.05]
Hoshimoto S 2019	0.5799	0.4142	4.6%	1.79 [0.79, 4.02]
Kitano Y 2017	0.2788	0.2435	9.8%	1.32 [0.82, 2.13]
Lin GH 2016	0.8156	0.3055	7.3%	2.26 [1.24, 4.11]
Ma B 2021	0.733	0.3	7.5%	2.08 [1.16, 3.75]
Omichi K 2017	0.2427	0.2572	9.2%	1.27 [0.77, 2.11]
Zhang Y 2019	0.107	0.1729	13.8%	1.11 [0.79, 1.56]
Zhang ZY 2020	0.0186	0.0373	22.9%	1.02 [0.95, 1.10]
**Subtotal (95% CI)**			**100.0%**	**1.33 [1.10, 1.62]**
Heterogeneity: $Tau^2 = 0.04$; $Chi^2 = 19.58$, df = 8 (P = 0.01); $I^2 = 59\%$				
Test for overall effect: Z = 2.88 (P = 0.004)				

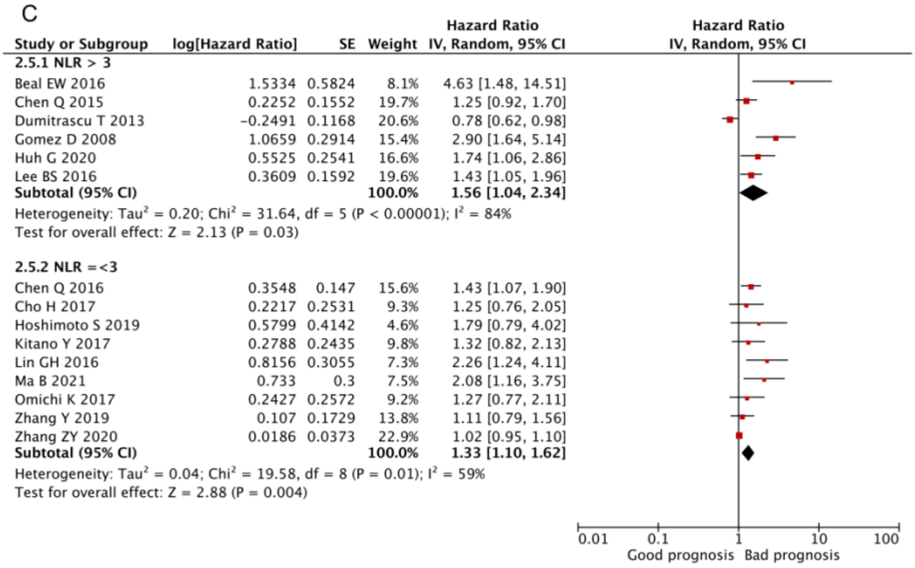

B

Study or Subgroup	log[Hazard Ratio]	SE	Weight	Hazard Ratio IV, Random, 95% CI
**2.2.1 Surgery**				
Beal EW 2016	1.5334	0.5824	2.0%	4.63 [1.48, 14.51]
Chen Q 2015	0.2252	0.1552	8.6%	1.25 [0.92, 1.70]
Chen Q 2016	0.3548	0.147	8.9%	1.43 [1.07, 1.90]
Dumitrascu T 2013	−0.2491	0.1168	9.7%	0.78 [0.62, 0.98]
Gomez D 2008	1.0659	0.2914	5.2%	2.90 [1.64, 5.14]
Hoshimoto S 2019	0.5799	0.4142	3.3%	1.79 [0.79, 4.02]
Kitano Y 2017	0.2788	0.2435	6.3%	1.32 [0.82, 2.13]
Lin GH 2016	0.8156	0.3055	4.9%	2.26 [1.24, 4.11]
Ma B 2021	0.733	0.3	5.1%	2.08 [1.16, 3.75]
Zhang Y 2019	0.107	0.1729	8.1%	1.11 [0.79, 1.56]
Zhang ZY 2020	0.0186	0.0373	11.4%	1.02 [0.95, 1.10]
**Subtotal (95% CI)**			**73.5%**	**1.40 [1.13, 1.74]**
Heterogeneity: Tau² = 0.08; Chi² = 45.24, df = 10 (P < 0.00001); I² = 78%				
Test for overall effect: Z = 3.03 (P = 0.002)				
**2.2.2 Non-surgery**				
Cho H 2017	0.2217	0.2531	6.0%	1.25 [0.76, 2.05]
Huh G 2020	0.5525	0.2541	6.0%	1.74 [1.06, 2.86]
Lee BS 2016	0.3609	0.1592	8.5%	1.43 [1.05, 1.96]
Omichi K 2017	0.2427	0.2572	5.9%	1.27 [0.77, 2.11]
**Subtotal (95% CI)**			**26.5%**	**1.42 [1.15, 1.75]**
Heterogeneity: Tau² = 0.00; Chi² = 1.07, df = 3 (P = 0.78); I² = 0%				
Test for overall effect: Z = 3.23 (P = 0.001)				
**Total (95% CI)**			**100.0%**	**1.39 [1.17, 1.66]**
Heterogeneity: Tau² = 0.07; Chi² = 52.53, df = 14 (P < 0.00001); I² = 73%				
Test for overall effect: Z = 3.67 (P = 0.0002)				
Test for subgroup differences: Chi² = 0.01, df = 1 (P = 0.93), I² = 0%				

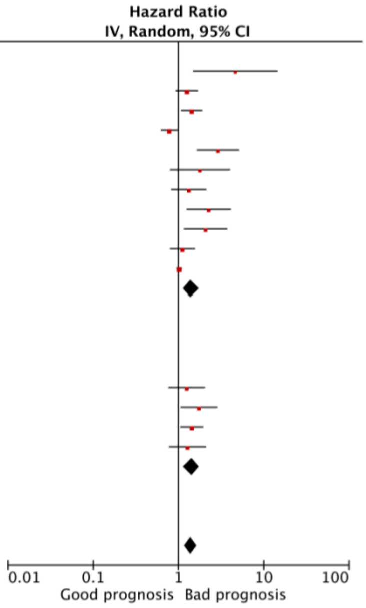

D

Study or Subgroup	log[Hazard Ratio]	SE	Weight	Hazard Ratio IV, Random, 95% CI
**2.4.2 Eastern**				
Chen Q 2015	0.2252	0.1552	11.6%	1.25 [0.92, 1.70]
Chen Q 2016	0.3548	0.147	12.0%	1.43 [1.07, 1.90]
Cho H 2017	0.2217	0.2531	7.1%	1.25 [0.76, 2.05]
Hoshimoto S 2019	0.5799	0.4142	3.5%	1.79 [0.79, 4.02]
Huh G 2020	0.5525	0.2541	7.1%	1.74 [1.06, 2.86]
Kitano Y 2017	0.2788	0.2435	7.5%	1.32 [0.82, 2.13]
Lee BS 2016	0.3609	0.1592	11.4%	1.43 [1.05, 1.96]
Lin GH 2016	0.8156	0.3055	5.6%	2.26 [1.24, 4.11]
Ma B 2021	0.733	0.3	5.7%	2.08 [1.16, 3.75]
Zhang Y 2019	0.107	0.1729	10.6%	1.11 [0.79, 1.56]
Zhang ZY 2020	0.0186	0.0373	17.9%	1.02 [0.95, 1.10]
**Subtotal (95% CI)**			**100.0%**	**1.36 [1.15, 1.61]**
Heterogeneity: Tau² = 0.04; Chi² = 26.33, df = 10 (P = 0.003); I² = 62%				
Test for overall effect: Z = 3.57 (P = 0.0004)				
**2.4.3 Western**				
Beal EW 2016	1.5334	0.5824	17.8%	4.63 [1.48, 14.51]
Dumitrascu T 2013	−0.2491	0.1168	29.6%	0.78 [0.62, 0.98]
Gomez D 2008	1.0659	0.2914	25.9%	2.90 [1.64, 5.14]
Omichi K 2017	0.2427	0.2572	26.7%	1.27 [0.77, 2.11]
**Subtotal (95% CI)**			**100.0%**	**1.72 [0.81, 3.63]**
Heterogeneity: Tau² = 0.48; Chi² = 25.69, df = 3 (P < 0.0001); I² = 88%				
Test for overall effect: Z = 1.41 (P = 0.16)				

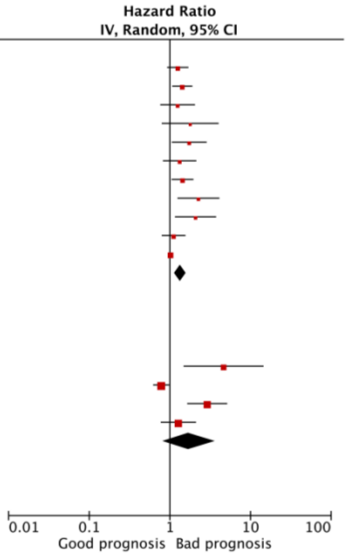

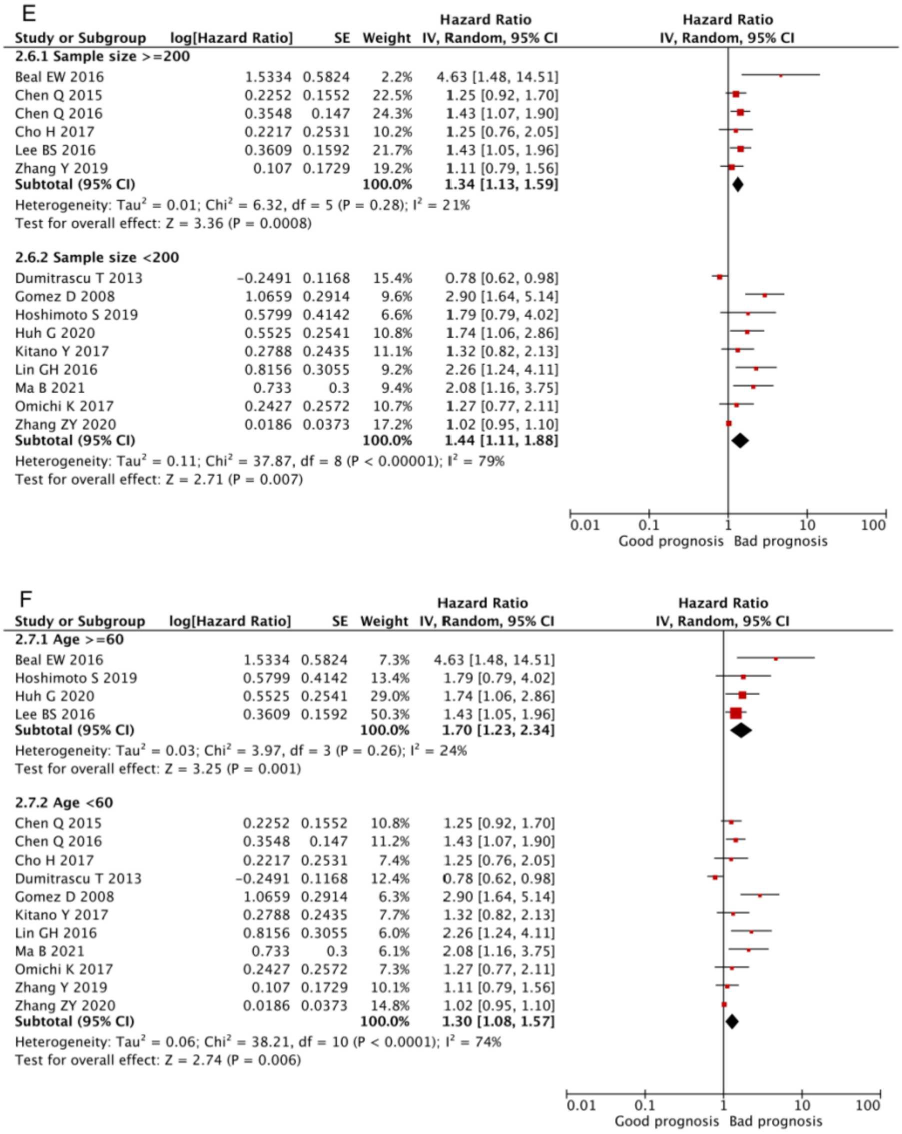

Subgroup analysis of association between the NLR and DFS based on cancer type (A), treatment (B), NLR cut-off values (C), region (D), sample size (E) and age (F). A random-effects model was used. CCA, cholangiocarcinoma; DFS, disease-free survival; ECCA, extrahepatic cholangiocarcinoma; ICCA, intrahepatic cholangiocarcinoma; NLR, neutrophil to lymphocytes ratio.

Figure S3 Baujat plot of the investigated data of overall survival (A) and disease-free survival (B).

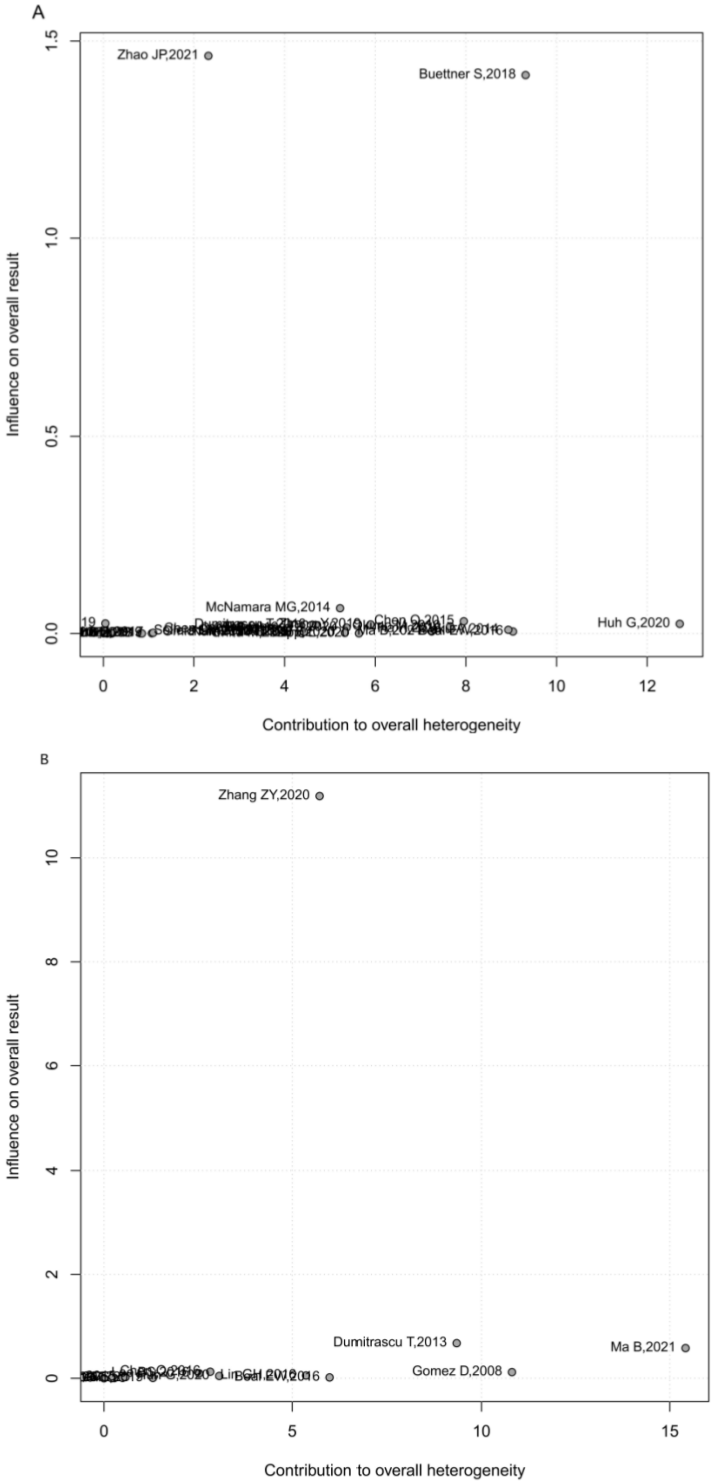

Baujat plots were used to assess contribution to the overall result and to heterogeneity. DFS, disease-free survival; NLR, neutrophil to lymphocytes ratio; OS, overall survival.

Figure S4 Funnel plots of the investigated data of overall survival (A) and disease-free survival (B)

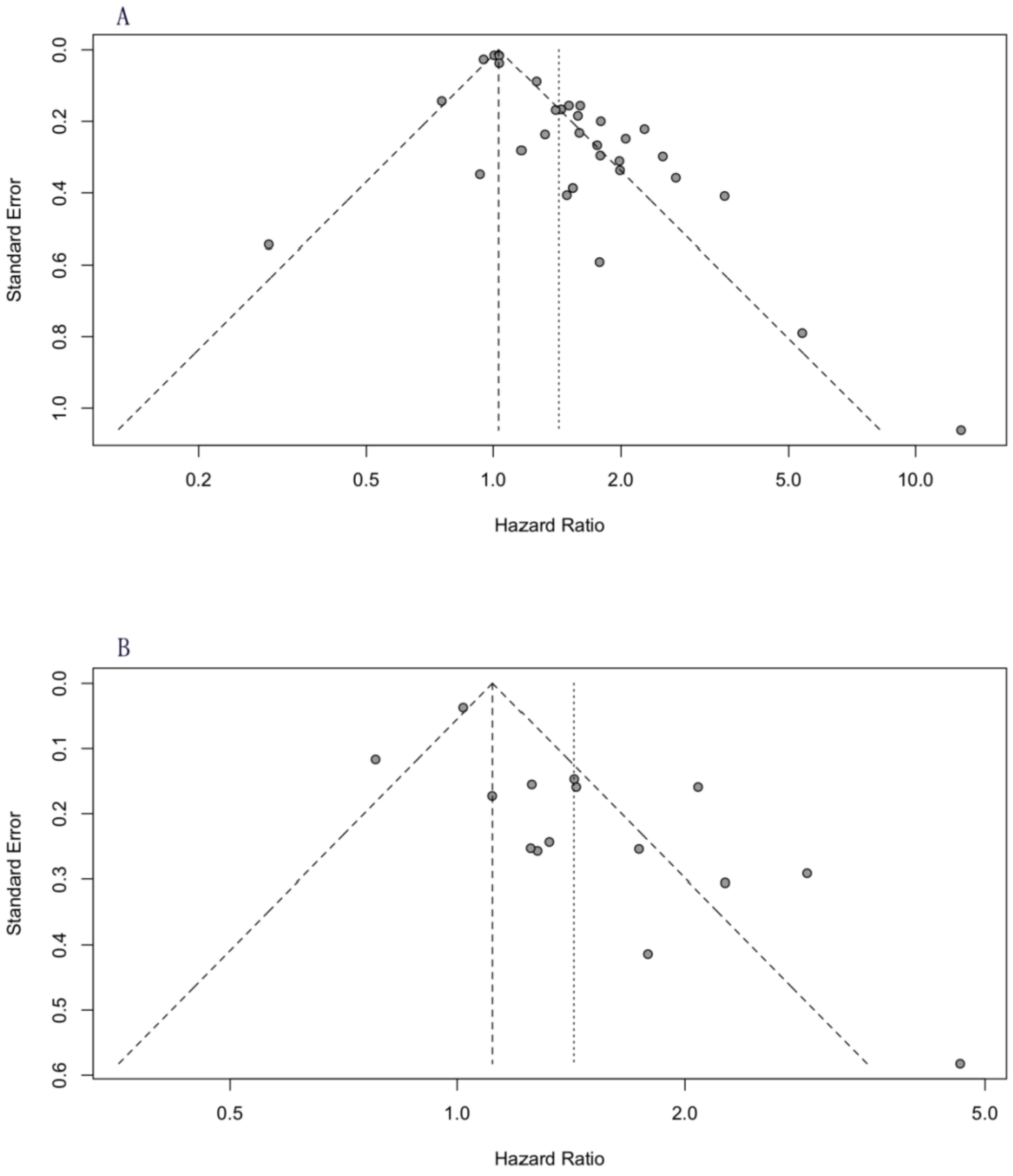

Supplement: Supplementary file 1 — Supplementary Information. [file 41598_2022_16727_MOESM1_ESM.pdf]
